# Performing Surgery: Commonalities with Performers Outside Medicine

**DOI:** 10.3389/fpsyg.2016.01233

**Published:** 2016-08-31

**Authors:** Roger L. Kneebone

**Affiliations:** Centre for Engagement and Simulation Science, Imperial College LondonLondon, UK

**Keywords:** performance, magic, puppetry, simulation, surgery, clinical practice, interdisciplinary research, surgery as performance

## Abstract

This paper argues for the inclusion of surgery within the canon of performance science. The world of medicine presents rich, complex but relatively under-researched sites of performance. Performative aspects of clinical practice are overshadowed by a focus on the processes and outcomes of medical care, such as diagnostic accuracy and the results of treatment. The primacy of this “clinical” viewpoint—framed by clinical professionals as the application of medical knowledge—hides resonances with performance in other domains. Yet the language of performance is embedded in the culture of surgery—surgeons “perform” operations, work in an operating “theater” and use “instruments.” This paper asks what might come into view if we take this performative language at face value and interrogate surgery from the perspective of performance science.

## Introduction

Modern surgery is not usually conceived of as performance. Yet surgery has a long history as a performative practice, inextricably bound up with wider landscapes of performance and spectacle which characterize its wider sociotechnical contexts (Bouchard and Mermikedes, 2016). The development of “modern” surgery takes place alongside changes in many forms of performance from the eighteenth century onwards, reflecting and responding to wider social currents which reverberate to this day. In the case of music, for example, a progressive professionalization which separated dilettante music-making from concerts for passive paying audiences continues to shape relationships between audiences, performers, and composers as they evolve (McVeigh, 2012). Similar currents take place within science. Scientific demonstrations, such as eighteenth century experiments with the air pump, condensing engine and electrical machine, established performance as a “public spectacle of natural philosophy” whose ripples continue into our own times (Schaffer, 1983). Similar developments affected other forms of performance, such as the puppetry and magic described below (Schechner and Appel, 1990; Francis, 2012; Lamont, 2013).

This performative aspect of surgery was evident in Britain and across Europe (Bynum and Porter, 1985; Lawrence, 1992; Jackson, 2001). The eighteenth century was a time when surgery was establishing a new identity. As Gelfand puts it, “In a metaphorical sense that comes close to being literally true, the [Parisian] surgeons […] may be thought of as sons of the medieval barber-surgeon and fathers of the modern physician-surgeon” (Gelfand, 1980). Performance in the form of demonstration—from public anatomies to operative procedures—became a key component of surgeons' claim to legitimacy as scientists as well as artisans.

Spary argues that in France especially, performance was central to the establishment of surgery as a high status profession, a means by which surgeons navigated their new territories (Spary, 1999). She too points out that eighteenth century surgery had a strong element of spectacle, often performed before a domestic audience. Surgeons had to negotiate the challenges of appealing to a sensation-seeking public while distancing themselves from charlatans. In her words, “surgeons gradually came to hide the mechanism behind their operative stage even from themselves. The making invisible of the social body required to perform the operation successfully was mirrored by surgeons' claims to make visible the invisible parts of the natural body in which they operated.” A maxim of the time—that “a true surgeon, a learned and experienced man, seeks to count his successes only by the operations he has known how to prevent”—resonates, as will be discussed below, with current issues around relationships between performance and professional judgment.

In the eighteenth century, as now, surgical performance played out on multiple levels (Leder, 1992). Guerrini, for instance, uses the example of Alexander Monro *Primus* (Professor of Anatomy at Edinburgh University) to explore the idea of the “moral theater of anatomy,” where dissection of human cadavers and live animals took place simultaneously in front an audience for whom “function as well as form was the object of study.” A constant tension is evident here, balancing entertainment, instruction, and clinical care (Sawday, 1995). While serious themes of death, repentance, and retribution were demonstrated though dissection of dead human and living animal bodies, the theater of anatomy was also “the ultimate entertainment […] In eighteenth-century Edinburgh, it was the best show in town” (Guerrini, 2006).

There is therefore a historical precedent for considering surgery as part of a broader performance canon. Returning to the present, if performance science is defined as a “multidisciplinary study of human performance which draws together methodologies across numerous scientific disciplines, including those of psychology, physiology, sociology, and economics, to understand the fundamental skills, mechanisms, and outcomes of performance activities and experiences” (Williamon 2016, under “Frontiers in psychology; performance science; scope”)^1^, there is much to be gained by applying its powerful approaches to surgical practice as an instance of performance activity and experience. The ambiguities of surgery (especially in terms of who is performing what, for whom and to whom, and who exactly is the audience) pose interesting challenges which invite fine-grained analysis.

So what constitutes surgery and in what ways might it be considered performance? A widely held lay view frames the operating theater as the primary site of surgical activity. This paper broadens that reading by locating the operation as part of a complex picture which encompasses consultation, diagnosis, and treatment. All these elements demand expert performance, though instantiated in different ways.

Such a framing involves shifting our viewpoint from outcomes (the “what” of surgery—removing this organ or treating that condition) to process (the “how” of surgery—its performance). This invites researchers to step back from the specifics of individual procedures and consider surgery as a domain of expert performance where the perspectives of audience and performer must both be accounted for. Yet clinicians are often skeptical of the notion of performance, seeing it in terms of “entertainment,” something that is not “real work.” The concept, unfamiliar to many clinicians, of a science of performance presents a legitimizing lens which invites new conversations and opportunities for collaboration.

To explore this tension, the paper places aspects of surgery alongside cognate domains of expertise outside medicine which are recognized as instances of performance. Two case studies, taken from an extensive body of exploratory work by the author, identify specific parallels. Exploration with magicians suggests viewing the clinical consultation as a “close-up live performance with a very small audience.” This highlights techniques for establishing initial rapport, managing attention, creating a relationship of trust and integrity, and constructing a retrospective perception of what has taken place. Exploration with puppeteers shines light on the practices of operative surgery, of “reading bodies within a dextrous team.” This highlights the physicality of hands and fingers, the challenges of working with transient teams, the need to develop awareness of others in a group, and the role of feedback and positive critique. Both magic and puppetry have of course evolved alongside musical and scientific performance, sharing many of their social contexts.

The paper poses the following questions:

1. To what extent and in what ways can surgical practice (both consultation and operation) be considered as performance?
2. How does comparison with two domains of non-surgical performance (close-up magic and puppetry) illuminate understanding of surgical practice as performance?
3. In what ways might including surgery within the canon of performance studies enrich the field of performance science?

## The clinical world

A brief description of two core strands of surgical practice—the consultation and the operation—sets the scene for the non-clinical reader.

### The consultation

The consultation lies at the heart of all clinical care. A clinician and a patient are held together in a relationship of care based on trust, integrity, and professionalism. Though apparently straightforward, the consultation is an example of the “art that conceals art” and shows many of the hallmarks of an expert improvised performance (Sudnow, 1978; Neighbour, 1987, 1992; Alterhaug, 2004; Krivis, 2006; Johnstone, 2007; Solis and Nettl, 2009).

Within each encounter, a skilful practitioner in any branch of medicine will establish a rapport with a patient whom they may never have met, eliciting key information by a combination of sensitive questioning, physical examination and the results of blood tests and imaging (such as radiographs and scans). While appearing to give the patient their undivided attention, the clinician will be carrying out a silent internal conversation, considering a range of possible diagnoses and treatments and selecting the most appropriate way of communicating based on their evolving assessment of the patient's level of understanding, anxiety and desire for knowledge. The clinician will formulate alternative options, discuss these with the patient and jointly agree upon a way forward.

Often this relationship is one to one. On other occasions, especially when the possibility of surgery involving children or family members is to be discussed, several people will be involved. Where an operation is being considered or planned, the risks, benefits, and potential complications will be explored. Similar aspects of consultation are seen in care before and after an operation, including preoperative assessment, ward rounds or high dependency units.

From a clinician's perspective, skilful consultation requires the integration of embodied ways of knowing with more formal conceptual frameworks populated by detailed factual knowledge and internalized “illness scripts” (Schuwirth, 2002; Bleakley et al., 2003; Charlin et al., 2007; Norman et al., 2007; Woods, 2007; Croskerry, 2009). Although skills of history-taking and clinical examination are of course taught in medical schools, they are usually framed as steps toward diagnosis and treatment rather than as aspects of performance.

### The operation

For many lay people, the word “surgery” conjures a cluster of masked and gowned figures huddled around an unconscious form on an operating table. A hushed silence is broken by occasional terse commands issued by the chief surgeon and the atmosphere is tense and expectant as the patient's life hangs in the balance.

The reality is very different. Most of the time surgical operations are routine, uneventful procedures carried out by a group of expert professionals who display exceptional team working skills as well as dexterity and precision. Although the lead surgeon carries ultimate responsibility for the patient, leadership is distributed within the team and the fine motor skills of individuals play out within a highly trained ensemble.

The nature of surgical performance is in flux, and established ways of doing are continually reconfigured by technical and sociocultural change (Weldon et al., 2015b). Until the 1990s, most major operations were “open” procedures carried out under general anesthetic. Organs were exposed directly though incisions into the body and team members' view was determined by line of sight. Often the primary surgeon and first assistant were the only members of the team who could clearly see the operative field. The team's engagement was with a depersonalized body, mediated through monitors and machines, and the “personhood” of the patient could be bracketed out until the operation was over (Goodwin, 1994; Whitty et al., 1996; Goodwin et al., 2005; Mort et al., 2005; Hindmarsh and Pilnick, 2007; Pope et al., 2007).

In the last 30 years, laparoscopic (keyhole) surgery has become the norm for many operations. Procedures are carried out through tiny incisions using long rigid instruments, and miniature cameras display a magnified image of the patient's anatomy on operating theater screens which all can see (Burkitt et al., 2009; Kneebone and Woods, 2014; Frampton and Kneebone, in press). Advances in anesthesia (such as local and regional blocks) mean that patients now are frequently conscious while undergoing major procedures. This is profoundly altering the dynamic between patient as person and patient as body.

The democratization of view described below means that patients (if they wish) can see on the screen what the clinical team sees, as well as being aware of the team around them. A progressive turn toward interventional radiology means that many procedures are carried out by means of flexible wires inserted at a distant site and steered into position under imaging control. Complex interventions on the heart or brain, for example, may be conducted through a tiny wire introduced through a distant artery in the wrist or groin. Paradoxically, as such procedures become more remote, the patient becomes more present.

This presence of the patient as a person (rather than a depersonalized body) during surgery invites a further re-examination of relationships between “audience” and “performer” (Kneebone, 2014). Clinicians must communicate with patients as they carry out delicate maneuvres, adjusting their attentional focus as the procedure evolves (Kneebone, 2013). This requires clinicians to construct a world where the patient (as audience) is willing to have their attention shaped in particular ways, to have anxiety relieved yet remain informed of what is happening, leaving with an agreeable retrospective memory of the experience.

Alongside these developments in the technical aspects of surgery, surgical teams are changing. Traditional notions of hierarchy, authority, deference, and control are being redefined. Many operations are performed by “transient teams,” whose members may meet for the first time at the start of a major operation. Each member is performing to others in the team, as well as to the patient (Bezemer et al., 2011a,b,c, 2013; Weldon et al., 2013, 2015a; Korkiakangas et al., 2014, 2015; Bezemer and Kress, 2016) Within such transient groupings, shared practices built up over years of working in a stable team can no longer be taken for granted and must be negotiated afresh on each occasion (Bezemer, 2009; Weldon et al., 2013; Cope et al., 2015; Korkiakangas et al., 2015, 2016; Snell et al., 2015).

Similarities between surgery and other kinds of performance include the following:

- An operation once begun must continue to its conclusion, whatever the difficulties or challenges along the way.
- High levels of fine motor skill are deployed by individuals working as members of a team.
- Surgery takes place before an audience, though it may not be easy to define who that audience is.
- Every operation is unique, unfolding in the moment and never wholly predictable.
- Successful surgery demands intensive training and preparation and the ability to develop reliable “automated” routines for straightforward procedures.
- Although surgery is generally extremely safe, there is always the potential for complications to arise and disasters occasionally happen.
- Performers must alternate their focus between extreme detail at the operative site (sometimes magnified through loupes or operating microscopes) and situational awareness of the bigger picture (encompassing the whole operating theater and beyond).

Differences between surgery and other kinds of performance include the following:

- The implications for “patient as audience” are radically different from the implications for other categories of audience (as concert or theatregoer, for example, where nobody dies or gets hurt from a fluffed line or a wrong note).
- Traditional theatrical practices of direction, stage management, feedback, and review are differently mediated within surgery and often absent altogether.
- Surgery is designed to take place in seclusion and is seldom explicitly framed as performance.

## Frames and frame-switching

For some clinicians, the notion that their work might be considered as performance can cause discomfort. Although the terminology of performance is enshrined in the everyday parlance of surgery (“performing” operations in an operating “theater” using surgical “instruments”), the primary focus of contemporary surgical activity is seen as the procedure itself—removing a cancer, treating a fracture, relieving an obstruction or repairing a leaking valve. Yet surgical insiders develop (whether consciously or not) sophisticated performance techniques as well as methods for managing anxiety and stress, for dealing with the consequences of error and for managing feedback and criticism from colleagues, patients and publics. These techniques have been developed largely within the world of surgery itself, with little reference to other literatures or performance traditions, and usually take place at an individual rather than a team level. This is a consequence of the surgical frame, which imposes a particular view upon those inside it, inviting them to compare themselves with other insiders but not to look beyond the frame itself. As with many experts, the practice of surgeons takes place within specific boundaries, furnished with and depending upon an insular body of knowledge (Atkinson, 2004; McConachie and Hart, 2006; Bleeker, 2008; Schechner and Brady, 2012; Williamon et al., 2014).

Goffman sheds light on frames, kinship relationships and the formation of professional identity (Goffman, 1974). Comparing oneself with others in one's primary field (e.g., surgery) both reveals and conceals relationships and connections. Within this frame, surgeons might see their “nearest relatives” as other kinds of doctor (such as anesthetists, physicians, psychiatrists) or more widely as other kinds of clinician (such nurses, physiotherapists, or operating department practitioners). A switch in frame—viewing surgery as a form of performance, for instance—brings different kinships into view, highlighting actors, dancers, musicians, or sportsmen as performing cognate work. Yet the historical traditions of clinical care and clinical education do not make such frame-switching easy. As a consequence, other performers' experience remains inaccessible to clinicians.

As with many expert domains, the surgical frame imposes considerable pressure to conform. Those wanting to become insiders try to emulate the behavior of those already inside (Taussig, 1993; Melberg, 1995; Auerbach, 2003). Novices seldom think to challenge the nature of the frame itself, as they are using their attentional resources to learn their craft and become accepted within their primary world of practice (Bereiter and Scardamalia, 1993; Bereiter, 2002). This restrictive framing is intensified by surgeons' preoccupation with acquiring the fine motor skills which their occupation demands.

Musicians, sportsmen, magicians, and other usually begin practicing the physical skills of their chosen field when very young (Ericsson et al., 1993, 2006; Ericsson and Charness, 1994; Williamon, 2004; Ericsson, 2007). By the time they come to perform in the adult world, they have had years of developing procedural memory. Outstanding embodied ability has marked such people out from an early age and forms the basis of their claim to possess exceptional talent. Surgical training is markedly different. Before trainee surgeons even start their specialist training they have spent perhaps 8 years since leaving school, undergoing undergraduate education, and early post-qualification attachments. Selection into medical school and then into postgraduate surgical training is based on evidence of cognitive performance rather than fine motor skill or psychomotor aptitude. At the start of their specialist training (in their mid-twenties, say), surgical trainees have hardly started to acquire the fine motor skills they will need as they develop. There is a strong cognitive emphasis, and skills lag far behind factual knowledge.

Many novice surgeons are acutely aware of their shortcomings in surgical technique. Their initial focus is often around acquiring these skills of the hand—which are not only essential for the practice of surgery but which also contribute greatly to surgeons' sense of identity (Mylopoulos and Regehr, 2007, 2011; Moulton et al., 2010a,b; Mylopoulos et al., 2011; Jarvis-Selinger et al., 2012) This preoccupation with the physical aspects of operating can overshadow awareness of interpersonal and other skills in the operating team. The wider “performance” of surgery takes longer to crystalize (Fry and Kneebone, 2011).

In surgery, most learning takes place through participation in operations, initially as a legitimate but peripheral participant in a community of practice (Lave and Wenger, 1991; Wenger, 1998; Guile and Young, 2001). In contrast with music conservatoires, for example, with their emphasis on incessant practice and rehearsal (though much less on the mechanics of performance), opportunities for practice within surgery are limited (Kneebone, 2009; Watling et al., 2014).

## Simulation as a research tool

In order to examine clinical practice as performance it is necessary for a researcher to access and experience it, at least at some level. Since much of surgery (as with expert performance more generally) involves tacit embodied ways of knowing that cannot be conveyed by words alone but are expressed through multiple modes, this presents a challenge (Bezemer and Kress, 2016). For obvious reasons of confidentiality, security and infection control, access to surgical activity is highly restricted. This inaccessibility of the originary world suggests a reason why surgery has been left out of discussions about performance.

The “insider” literature of surgery is of little help to non-clinicians, since everyday ways of doing are not documented there. Descriptions of operations seldom refer to the functioning of the operating team or to the taken-as-read context of professional practice at the time of writing (Burkitt et al., 2009; Williams et al., 2013). A knowledge of surgical practices is assumed as unproblematic, self-evident, and known to all practitioners. Since outsiders cannot attend a surgical performance, however, it is difficult for them to gain an authentic sense of the surgical world.

This section proposes simulation as an experimental setting for cross-disciplinary investigation. For centuries, simulation has provided a means for clinicians to gain practical skills without harming living patients. Owen's detailed and scholarly history shows that “machines” or “phantoms” were well-established by the eighteenth century (especially in obstetrical practice), and that medical simulation continued to flourish and develop during the centuries that followed (Owen, 2016).

Although simulation has now become a mainstay of clinical education, its resources until recently remained the province of insiders, a means by which surgeons, nurses, anesthetists and others could practice routine skills and emergency procedures with the safety of a simulation center (Gaba, 2004; Rall and Gaba, 2004; Dieckmann et al., 2007). A wide range of simulators, from physical models to virtual reality computer programs, are employed within medical schools and training centers across the world, allowing individuals and teams to gain skills without endangering actual patients (Owen, 2016). Simulated operating theaters, for example, are designed to recreate the originary world in every detail (though such facilities are scarce and costly).

A technicist focus, though necessary, is not sufficient. Unlike aircraft cockpits, where all Boeing 747s of a particular model are broadly similar, every person is unique. The work outlined below aims to place the patient “as person” (not only “as body”) at the center, and to allow for the uncertainty and ambiguity which characterizes clinical practice.

Three approaches to simulation developed by the author—initially designed for insider training—can create conditions for study by performance scientists, recreating conditions of surgical practice and making them accessible to “outsiders.”

### Simulating the consultation: hybrid simulation

**Figure 1:**
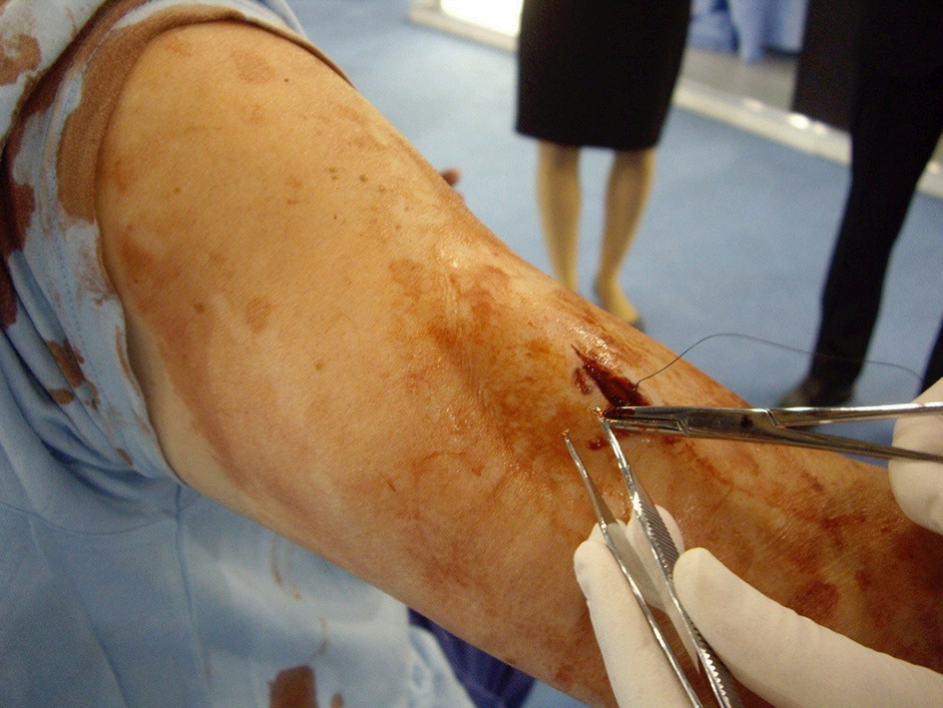
**Hybrid simulation of arm wound applied to Simulated Patient**.

**Figure 2:**
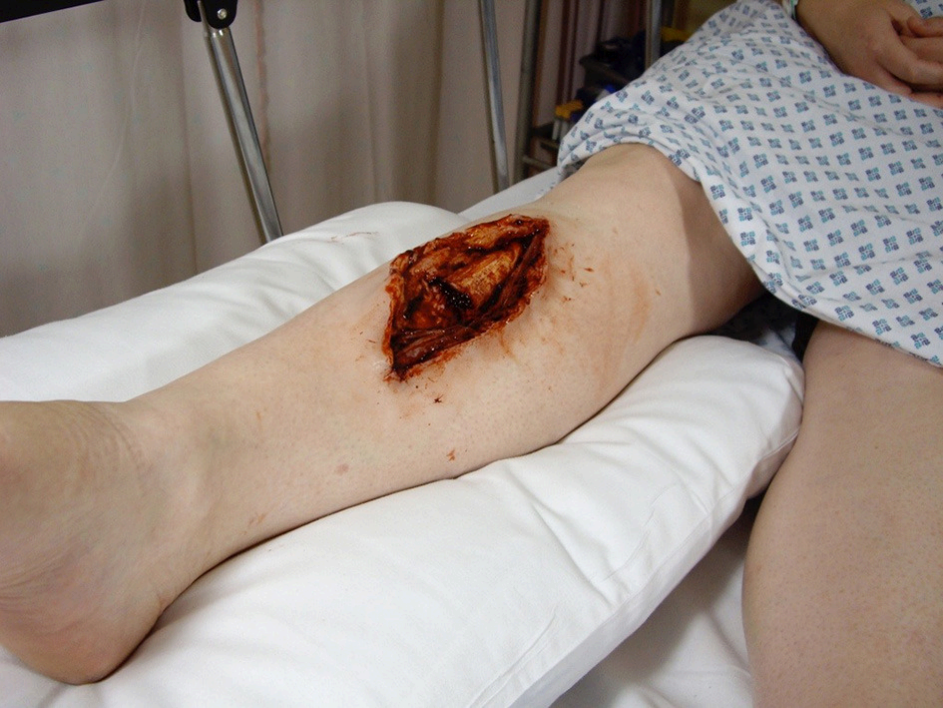
**Hybrid simulation of open leg fracture on Simulated Patient**.

Lifelike prosthetics applied to professional actors (Simulated Patients) in such a way as to conceal the join allow healthy humans to be modified so as to closely resemble sick or injured patients (Figures 1, 2). The presence of a real person recreates the conditions of a clinical encounter in a way that is impossible using inanimate models alone. Skilled actors portray the lived experiences of actual patients, preserving confidentiality while allowing the level of challenge to be adjusted at will (Kneebone et al., 2002, 2003, 2005, 2006a,b; Kneebone, 2006; Nestel et al., 2006).

### Simulating the operation: distributed simulation

**Figure 3:**
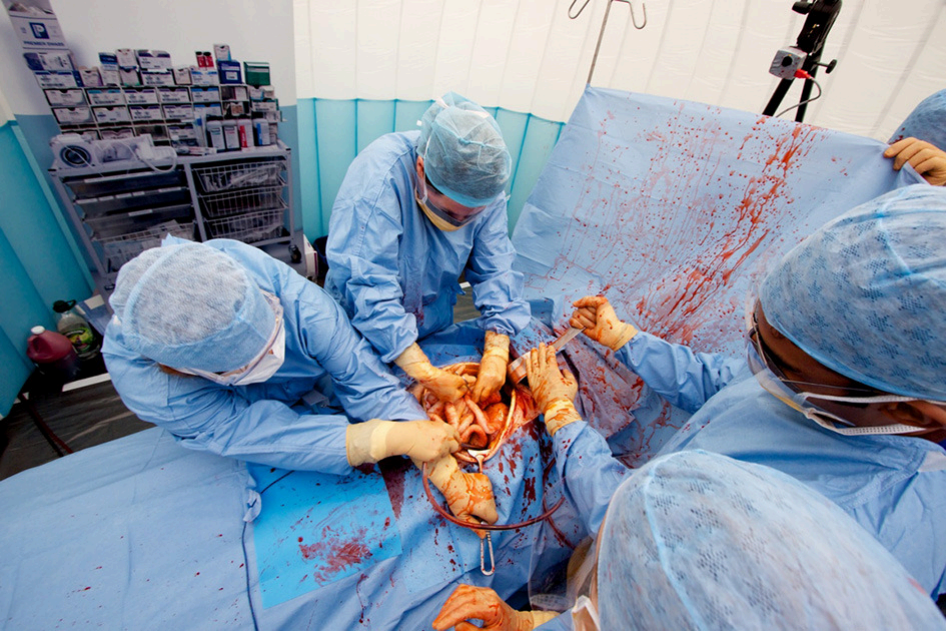
**Full team simulation of surgical operation**.

**Figure 4:**
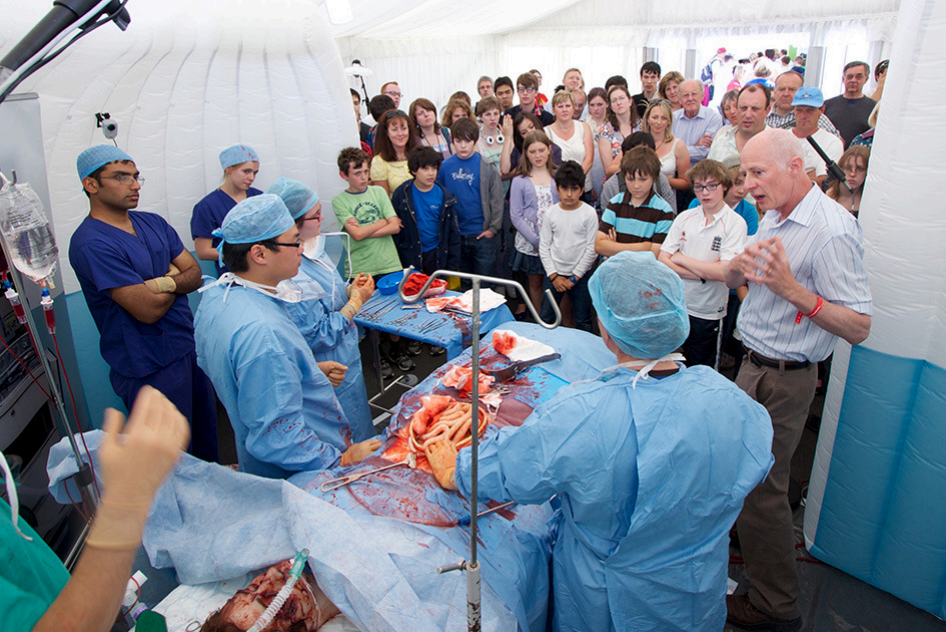
**Simulated operation with public audience at Cheltenham Science Festival**.

Portable, low-cost simulation provides realistic yet affordable environments within which clinicians can carry out surgical procedures. Theatrical backdrops and portable “flats” provide recognizable landmarks of each aspect of care (such as ward, operating theater or intensive care unit). Silicon models of internal organs allow procedures to be carried out, including exploration of the abdominal cavity, staunching of bleeding and the removal and rejoining of tissues using real surgical instruments (Figures 3, 4). This allows non-clinicians to “scrub in” with an operating team, experiencing many of the sensations and challenges of operating with a team of clinicians (Video 1) (Kneebone et al., 2010; Kassab et al., 2011, 2012).

### Simulating the system: sequential simulation

**Figure 5:**
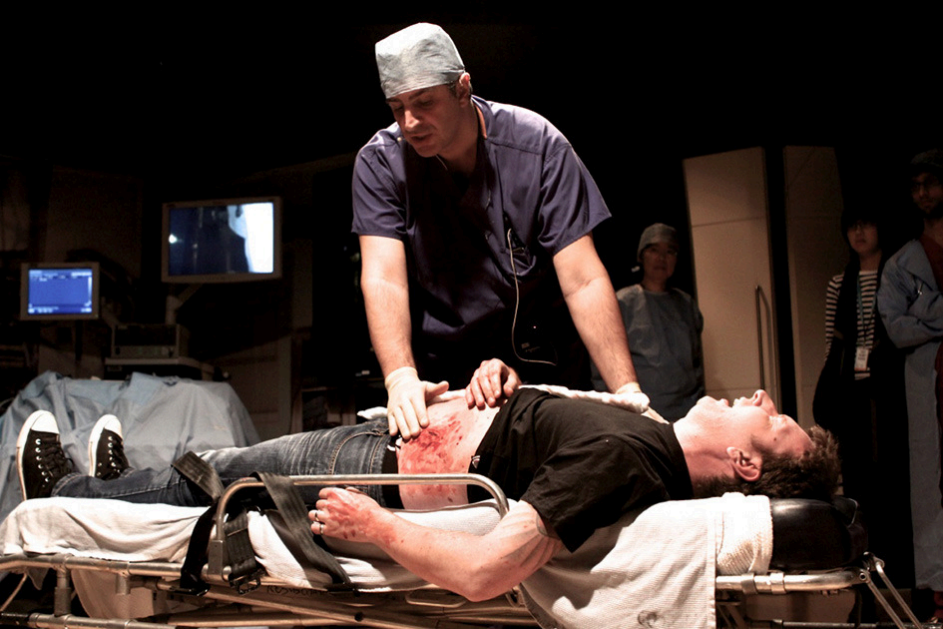
**Surgeon examining Simulated Patient with hybrid simulated stab wound**.

**Figure 6:**
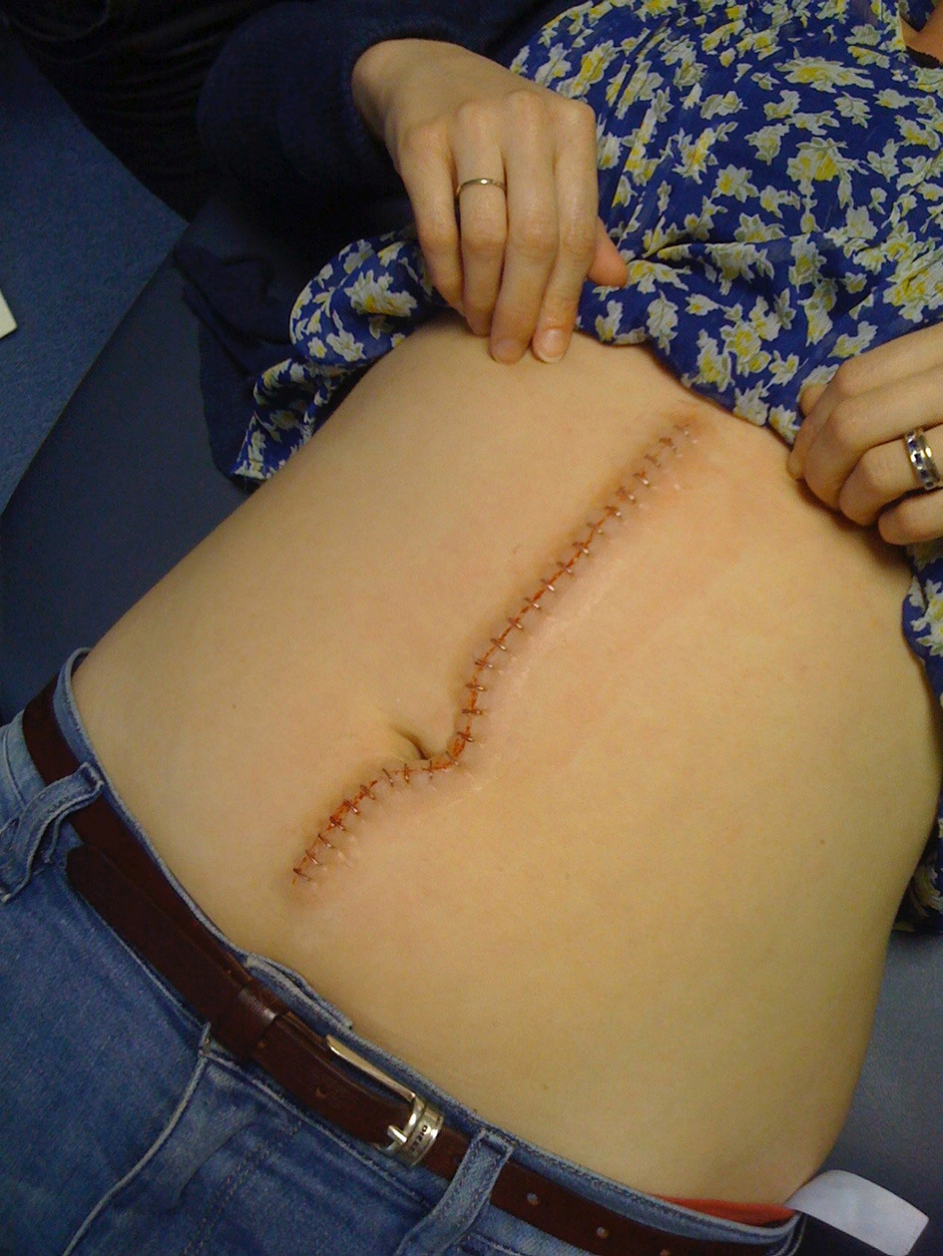
**Hybrid simulation of postoperative abdominal wound closure**.

A sequence of events is simulated, using “snapshots” of a care pathway to represent a patient's home, a GP's consulting room, an ambulance, an operating theater or a postoperative ward. A combination of Hybrid and Distributed simulation recreates the trajectory of a patient, allowing the broader world of surgery, both in the clinic and the operating theater, to be mapped and modeled (Figures 5, 6) (Weldon et al., 2015a, 2016; Video 2).

### Opening up to wider publics and other groups

**Figure 7:**
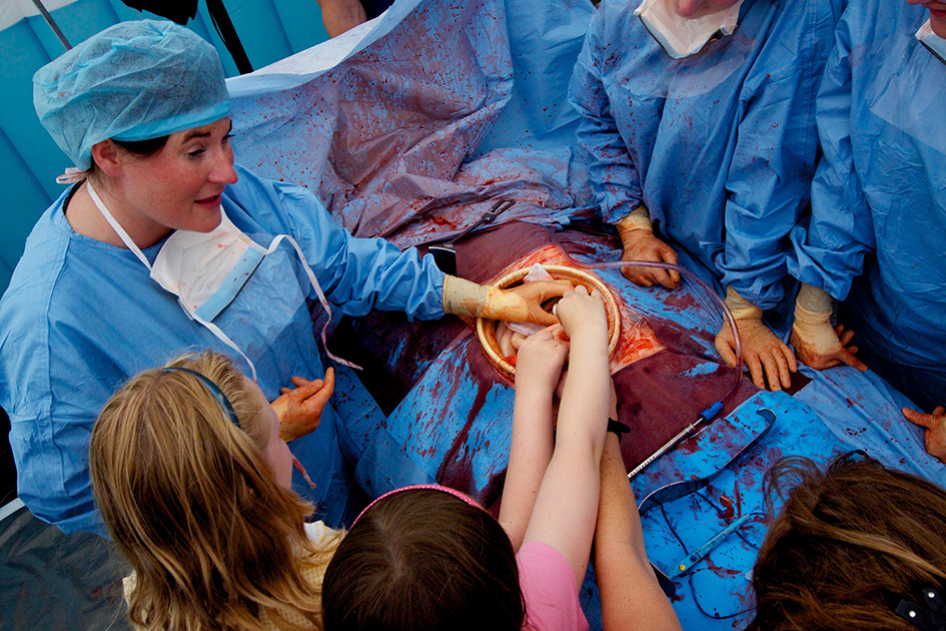
**Surgeon explaining operation to public audience at Cheltenham Science Festival**.

The author's group has used simulation of this kind to connect with worlds outside medicine. This offers new possibilities for dissolving the restrictive frame that normally bounds clinical practice and simulation-based learning. While recognizing the centrality of technical mastery and fine motor skills, this approach also highlights the social aspects of performance, ensuring that living human beings constitute the center. Over the course of 10 years the author has developed simulation of this kind in over 100 public engagement and other events, creating performance settings custom-designed for specific collaborations (Figure 7) (Tang et al., 2013).

The data presented in this paper consists of two detailed case studies drawn from an extensive body of exploratory work with performers and craftsmen outside surgery, carried out within the author's research group over an extended period. A broad corpus includes collaborations with performers in dance, jazz, classical music, magic, puppetry, juggling, theater and Formula One motor racing. This large body of data was generated with the aim of identifying exploring parallels between surgery and other areas of practice (craft and performance).

Two extended case studies form the experimental nucleus which follows. The aim is to offer lenses through which to observe clinical practice and to identify and characterize those aspects of surgery which may usefully be considered “performance.” Although no single area of performance exactly mimics or reflects the world of medicine, the following studies highlight aspects which are both relevant to clinical practice and have been highly developed outside it.

## Methods

### Data collection

This paper focuses in detail on two areas of expert performance—close-up magic and puppetry. Each case study involved a shifting cast of participants, built around evolving discussions with key interlocutors. Each case study involved a series of collaborative encounters with surgeons and interested academics (students and staff). Similarities and differences with surgery provided an initial point of contact and an enduring thematic focus. Simulation of surgical procedures (as described above) provided a common point of shared experience, allowing connection at the level of enacted practice (“doing” as well as “telling”).

Encounters were documented in detail combining field notes with audio and/or video recordings. Individual and group interviews and discussions were transcribed verbatim. Transcripts were checked against source recordings for accuracy.

### Data analysis

The analytical approach selected was thematic analysis, aimed at “identifying, analyzing, and reporting patterns (themes) within data” (Braun and Clarke, 2006). A phased approach progressed through familiarization with the data, generation of initial codes, searching for themes, reviewing themes, defining and naming themes and generating a report.

Data were subjected to repeated listening and review. Thematic analysis was performed initially within each data set (magicians and puppeteers). Data were collated and initial coding was carried out. A provisional thematic structure (identifying selected themes and sub-themes) was developed and refined through repeated review.

Themes were developed iteratively and progressively refined over the course of the research collaborations, aiming to capture levels of “patterned response or meaning” (Braun and Clarke, 2006). Each data set (magic and puppetry) was treated separately at first. In the later stages of the process the data sets were examined against the surgical context and discussed with surgical colleagues, identifying areas of similarity, and difference and shaping concepts relating to performance in the context of surgical practice. The aim at this stage was to identify aspects of these performances that might resonate with surgery, without pre-judging what might emerge. Discussions within the author's research group and with wider groups of colleagues and publics (general and selected) honed and narrowed the themes (Appendix in Supplementary Material).

Results from each case study are presented below, using selected verbatim quotations to support primary and secondary themes from the thematic analysis. Key quotations are provided in the body of the text in order to contextualize the themes, while more extensive and additional quotations are provided in the Appendix in Supplementary Material (using the same thematic structure). The Discussion section explores connections between these data sets and the practices of surgery.

### Close-up magic

Over a 60 month period, eight professional close-up magicians took part in the study. A total of 71.5 h of contact time include participative events, discussion sessions, and focused conversations (private and public) around surgery and magic.

*Phase 1* (January 2011 to June 2014): Initial explorations with individual performers (both in the UK and USA) were expanded through group discussions within the Imperial College London Masters in Education (M Ed.) in Surgical Education (designed and led by Kneebone).

*Phase 2* (June 2014 to December 2014): In depth exploration with an international group explored similarities and differences between magic and surgery. A full-day closed-door meeting in London in December 2014 between six magicians, three clinicians (including the author), a semiotician and other researchers provides a primary reference point for this study. The participating magicians (all members of the Magic Circle or its American equivalent) are highly regarded professionals in their field with successful careers in the field of magic, and all are recognized by their peers as outstanding performers. Discussion took place under the Chatham House Rule to ensure confidentiality. This was especially important in the context of professional magicians, allowing them to share details of their individual practice without anxiety about disclosing “trade secrets.”

*Phase 3* (January 2015 to Present): Eight formal discussion events (both private and public) in the succeeding months investigated in more detail issues that had emerged from the initial group meeting. These included further educational events with surgical consultants, trainees and other clinicians, and two public discussions with magicians and surgeons held at the Wellcome Collection in London. These too were captured by video and/or audio recording.

Member checking through further interviews with participants allowed emerging understandings to be tested and refined. Verbatim quotations within the themes are used to ground and contextualize the discussion. Quotations are anonymized, referring to experts by number.

The primary theme to emerge from the analysis was of magic as a “close-up live performance with a very small audience.” This distinguished close-up magic from stage magic performed to large audiences. A key characteristic of close-up magic was the need continually to engage with audience members as individuals, integrating performance with their responses.

Three secondary themes (with sub-themes) were as follows:

**1 Magic as social performance**
  - gaining attention
  - maintaining attention
  - closing the performance
**2 Magic as motor performance**
  - gaining manipulative skills
  - integrating skills within performance
**3 Magic as an internal process for the magician**
  - “the shift from you to them”
  - awareness of and sensitivity to the audience
**1 Magic as social performance**
  Close-up magic is jointly constituted by audience and performer.
  *Magician 2:* “Magic happens in this gap between my eyes and your eyes, my mind, and your mind.”
  The performance itself is highly designed and constructed and its essence is a live encounter where unpredictability is a key element. This requires high levels of skill in establishing and shaping relationships with audience members. The following elements can be identified.
**1 a) Gaining attention**
  Successful magicians have developed precise and reproducible techniques for establishing rapport with their audience and generating a conducive atmosphere. This starts by creating a personal connection.
  *Magician 2*: “The first thing I would always do is shake someone's hand. That does two things. One, it says we're equal. The other thing, absolutely crucial, it says—“look me in the eye.” And at that moment I go “the game's started now,” because then I can make you look wherever I want, I can place your attention where I need it.”
**1 b) Maintaining attention**
  In addition to gaining their audience's attention, magicians must be able to direct that attention precisely. Eye contact, physical interaction and “body-reading” are crucially important.
  *Magician 2:* “In magic the face and the eyes are the most important. As a magician I need to know what's happening with the eyes. I have to know what you're thinking and to know that you know what I'm thinking at any moment.”
  Magicians emphasize that, contrary to popular belief, their art does not involve hiding things. Rather it requires the building of alternative perceptual worlds.
  *Magician 1*: “Magicians use the word “misdirection.” But actually it is really directing. Beginning magicians think it's about taking stuff away. But you can't really hide stuff. What you can do is build things.”
  Performers are aware of the fragility of the relationships they construct and the need to perform with integrity.
**1 c) Closing the performance**
  Magicians pay close attention to creating a shaped and constructed perception in their audience. This recollection may not correspond with what was actually said or done by the magician, since recollections are notoriously plastic and subject to subsequent refashioning.
  *Magician 5*: “Maybe the same analogy, even, works with a consultation, where you can say there are two versions of what a consultation is. One version is the thing that the person actually gets. The other version is the thing that they tell their husband or wife about when they get home, and they say, “How did it go?.””
**2 Magic as motor performance**
  Highly developed fine motor skills are an essential element of close-up magic. Acquiring these skills entails years of unremitting effort.
**2 a) Gaining necessary skills**
  Expert performances requires mastery of technique, so that this becomes automatic.
  *Magician 2*: “You need to spend as much time as possible doing this [mimes manipulation] and free up as much as possible of your brain capacity, as the one thing you can't do as a magician is be talking to you and then think “wait a minute, I have to get my little finger here …” [gestures]. The hands have to know how do to that, so that you have maximum opportunity to be doing the occupation stuff.”
  For successful magicians, gaining and maintaining such mastery is a satisfying experience in its own right.
**2 b) Integrating skills within performance**
  These skills must become so much second nature that nobody watching is aware that they even exist.
  *Magician 4:* “Part of what you're talking about is acquiring an unconscious mastery, being able to handle things to a high degree of precision without thinking consciously about what you're doing.”
  Magicians develop approaches to practice which allow them to maintain high levels of motor precision and accuracy.
  *Magician 3*: “Magicians fiddling with cards is the “automatic practice” that they do underneath their own radar. Kind of unconscious practicing that results in muscle memory.”
  These manipulative skills are only part of the wider context of performance—an awareness which comes with maturity as a performer.
  *Magician 4:* “This ability to apply fine motor skill within performance is something that only some magicians successfully master.”
**3 Magic as an internal process**
  Becoming a magician (as opposed to someone who can perform tricks) involves maturation and requires a perspectival shift that does not always occur.
**3 a) The shift from you to them**
  Several of the magicians in this study pointed out that “it's not about *you* [the performer], it's about *them* [the audience].” This apparent truism underlies a key transition point in becoming an expert performer—a shift in focus from the performer to the audience (the jointly constructed space between audience and performer).
  *Magician 3:* “Many conjurers go the wrong way. They think it's more about how they're doing it, or it's about themselves.”
**3 b) Awareness of and sensitivity to the audience**
  The creation of a shared frame between performer and audience requires collaboration whose essence is a personal relationship.
  *Magician 5:* “I think there has to be two people, and one person is doing one set of actions, which he or she has created in such a way that they're deliberately misinterpreted by the other person in a specific way.”
  Magicians are processing information on many levels at the same time, and taking action accordingly. This involves self-awareness and the ability to “sit on one's own shoulder” while performing.
  *Magician 2:* “You have to be ruthlessly observing yourself while you're performing.”
  Judgment is key, and such judgment only comes through long experience.
  *Magician 2:* “A really good magician knows when not to perform.”
  This aspect of performance requires an acute sensitivity to social interactions, allowing a performer to “read” a situation within a few seconds. Close-up magicians in particular recognize this, developing the ability to make and act on rapid social judgments.
  *Magician 2*: “Approaching a table of ten, you have to do that with 10 people almost instantly. Have to know who's head of the table, who's flirting, who's arguing …You're potentially interrupting five conversations. So you've got go over there as if you've got something more important to say or do than the people who're sitting there, having a very nice meal, didn't ask for a magician—so you have pick your exact moment, where you're going to stand—so if you are head of the table and I stand next to you—all of those geographical things—in seconds.”
  This requires constant awareness of audience dynamics and interaction. Magicians are in a state of high alertness as they perform, registering minutiae of audience response.
The second case study addresses a different kind of live perspective.

## Puppetry

Over a 30 month period, 11 puppet professionals (including a director and dramaturg, puppeteers and performers, makers, and wranglers) took part in the collaboration. A total of 50.5 h contact time included 15 participative encounters.

*Phase 1*: (May 2013 to August 2014): Explorations using surgical simulation demonstrated clinical practice in the consulting room and the operating theater. Surgical team members demonstrated open and laparoscopic (keyhole) surgery, using Distributed, and Sequential simulations in the author's research laboratory (as described above) to show individual and team work and explore its relevance to puppetry. Surgeons subsequently visited puppet theater space and workshops.

*Phase 2*: (February 2014 to Present): Evolving ideas were explored with invited groups of professionals from fields related to, though distinct from, puppetry. A formal collaboration led to the design and performances of “Who Pulls the Strings?,” an interactive event in a temporary marquee at Einsteins' Garden (part of the Green Man Music Festival) by clinicians and puppeteers. Ten further events (each between 1 and 3 h) include simulation-based clinical education and a range of crafts involving the management of threads (marionettes). In-depth discussion with surgeons continues to explore parallels.

Member checking through further interviews with participants allowed emerging understandings to be tested and further refined. As above, verbatim quotations within the themes are used to ground and contextualize the discussion.

The primary theme to emerge from this analysis was of puppetry as a performance based on “reading bodies within a dextrous team.” A key characteristic of puppetry was the delicate manipulation of objects by individuals working in concert with others. This process is underpinned by high levels of awareness of others in the team, and an ability to respond collaboratively to the unexpected during performance.

Two secondary themes (with sub-themes) were as follows:

**1 Puppetry as group performance**
  - establishing cohesion as a group
  - group warm-up procedures
  - expectations and role of critique and feedback.
**2 Puppetry as motor performance**
  - manipulation of strings and rods
  - finger and hand warm-up procedures
**1 Puppetry as group performance**
  Puppetry highlights performance aspects of a shared activity based around dextrous manipulation. Communication takes place at an embodied level, through “reading” one another's bodies. This sensitivity to the bodies and intentions of other performers has to be deliberately developed.
**1 a) Establishing cohesion as a group**
  Puppeteers are acutely aware of their dependence on one another as they perform.
**1 b) Group warm up**
  Puppeteers often expected to form newly constituted groups composed of unfamiliar colleagues. Physical preparation plays an important role.
  *Puppeteer 1:* “Warming up together is crucially important in getting in tune with one another.”
  This is accepted as normal within the puppetry community. Puppeteers take pains to establish an effective group relationship, even (perhaps especially) if they do not know one another.
  Preparatory exercises are seen as especially important in a performance genre where fine motor control is of primary importance and where performers are expected to maintain unnatural postures for extended periods (Video 3). Exercises may include passing a puppet or an imaginary shape from person to person. Puppeteers deliberately foster a sensitivity to the physical signals they receive from one another while performing,
  Team-working is clearly understood by all performers in a group. This is especially evident in Bunraku, an ancient Japanese form where three performers manipulate a single puppet. In contrast to marionettes, which are manipulated by strings, Bunraku requires the puppeteers to move their puppet directly. One (traditionally the most experienced) controls the puppet's head; another its back and arm; while a third controls the feet (Video 4). Although the puppeteers make no attempt to hide, remaining in plain view throughout, their focus as a group creates a powerful illusion for the audience that the puppet is alive and its manipulators are invisible. Although all three puppeteers are indispensable, there is an understood hierarchy within the trio. Traditionally, the puppeteer controlling the head is in overall charge.
  *Puppeteer 1:* “It's led by the head—who has to have a plan of what to do and where to go, even (especially) if improvising. Hand and feet puppeteers follow. Though the actual action may be led by another (like the feet when walking), the intention to act is determined by the head puppeteer.”
**1 c) Expectations of critique and feedback**
  Puppeteers expect and rely upon critique to improve and refine their work, taking it as entirely normal. Such feedback forms an integral part of the performance culture of puppetry.
  *Puppeteer 1:* “Feedback absolutely underpins every part of our process, it's built into our process. And that can actually be really hard, because it can be very personal, very direct, very cutting. We have feedback during our rehearsal process. And that feedback is generally speaking from a director; often amongst yourselves; anybody who comes in and watches the work; and your self-criticizing things of course—you're your own worst critic […] There's constant feedback.”
**2 Puppetry as motor performance**
**2 a) Manipulation of strings and rods**
  Puppeteers have an intense relationship with material objects as they perform.
  *Puppeteer 1:* “Puppetry is bringing life or movement to an object or material.”
  A focus on dexterity and sensorial awareness highlights the intersection between hands, instruments and materials and the ability to respond to subtle physical signals.
**2 b) Finger and hand warm-up**
  Finger and hand warm-up here serves a different function from the group warm-up exercises described above, with their emphasis on communication and “getting in tune” with one another. In this case warming up is seen as essential for achieving the highest levels of fine motor control by hands and fingers over strings, rods or other objects (Video 3).
  *Puppeteer 1:* “Most actors and dancers don't have much focus on hands at all. They'll focus on back or legs or whatever. But for puppeteers very particularly it's hands […] Same way as a sportsperson will warm up for a race, so they don't pull a muscle and they're ready to perform.”

## Discussion

The aim of this paper is to offer lenses through which to examine clinical practice and to characterize those aspects of surgery which may usefully be considered “performance.” An exploratory approach, based on the discussions which formed an integral part of the case studies outlined above, has identified aspects of close-up magic and puppetry which resonate with the world of surgery. Although no single area of performance exactly mimics or reflects the world of medicine, each of the studies highlights aspects which are relevant to clinical practice and have been highly developed outside it. The following discussion explores how the themes and sub-themes identified above illuminate the practices of surgery and constitute a case for including it within the canon of performance science. The discussion addresses the consultation and the operation as distinct yet mutually dependant domains of performance.

### Surgery as a “close-up live performance with a very small audience”

The clinical encounter can be viewed as an instance of close-up performance. When successful, each patient is wholly convinced by the effortlessness of the encounter and the authenticity of the clinician's attention. As with close-up magic, clinical consultations take place on an intimate scale, with performer and audience sharing a space which appears “social” rather than overtly performative. Both involve an encounter which does not appear to be scripted but evolves naturally within a conversational setting. Both require performance within a range of contexts, often outside the control of the performer (a hospital outpatient department, say, or a hotel ballroom where table magic is to be performed after dinner). Yet both are in fact meticulously designed, presenting an illusion of spontaneity through painstaking practice. The consultation can therefore be framed as an artful construction within an “engagement space,” jointly constituted by clinician and patient. This is a complex process which takes place on multiple levels.

Expert clinicians integrate history-taking, physical examination and the internal processes of diagnosis with expert presentation, modulated by an emerging awareness of the patient's concerns and expectations and an ability to “listen with all your senses” (in the words of a participating GP). This entails a shift in focus by the performer, moving from an internal to an external locus of attention. Continual recalibration is needed, acknowledging the audience rather than the performer as the central point. Magicians identified the need for a similar shift in focus when they pointed out that “it's not about you [the performer], it's about them [the audience].” This shift from “transmitting mode” to “receiving mode” on the part of the clinician receives relatively little attention within the medical literature yet is an essential element in effective care (Launer and Lindsey, 1997; Tamariz and Lehn, 2007; Rubio et al., 2015).

The consultation can therefore be framed as a jointly constituted performance with a very small audience (often of only one) between clinician and patient(s). It requires commitment by all parties in the construction of a shared experiential world. As with magic, medicine happens in a space between patient and clinician. If there is no patient, there is no consultation. It is not enough to have medical knowledge or skill. Such knowledge only becomes meaningful through performance. This process is not a simple transfer of information but requires a tailor-made account to be constructed with each individual patient, highlighting some aspects and downplaying others. Magicians, like clinicians, pay close attention to such construction.

As highlighted above, magic is not about concealing but about building an alternative universe—not hiding what is there so much as building up an experiential world. Experienced clinicians too are well-aware of their role in shaping expectations and of the consultation as an additive, not a subtractive, process. For example, if they ask a patient “how comfortable does that feel?” as they are examining a swollen joint, the response is likely to be very different from “how much does that hurt?.” This creation of a shared positive frame requires collaboration. Although in clinical practice the aim is not deliberate misinterpretation, there are clear similarities in terms of performance intention and technique (Wilson, 2014). To be successful, each phase (initiating, continuing, concluding) of the consultation entails specific performance techniques which can be learned and taught. In all of these, attention management is key (Macknik and Martinez-Conde, 2010). The ability to connect with every patient, whether new or already known, is a core skill, and constructing a successful engagement space requires high levels of self-awareness by a clinician. As with magicians, for whom individual “tricks” are tools in the service of a broader performance, the consultation is not primarily about the transfer of medical knowledge but about establishing a personal relationship.

In surgery and in magic, then, the first few moments of any encounter exert a profound effect upon the rest of the performance. Initial contact and establishing rapport are crucial. In the surgical context this involves assessing each patient's state of mind and responding appropriately from the outset, a process which requires close observation and high levels of alertness. Although seemingly natural and unforced, effective consultations embody consummate skill based on years of practice (Lamont, 2013).

Successful magicians have developed precise and reproducible techniques for establishing rapport with their audience and directing attention where they want it. Yet clinical training, especially in its early stages, is dominated by the need to acquire and recall factual knowledge, overshadowing the need to embed such knowledge with effective performance. The consultation requires rapid interpretation of non-verbal cues and immediate reaction to the nuances of a patient's response—all within an apparent relaxation and informality that belies the complexity of what is going on underneath (Launer, 2007). Eye contact, physical interaction and “body-reading” are crucially important.

Underlying this relationship between audience and performer is a process of collusion between performer and audience, whether conscious or not. In the case of a clinical consultation, this aims to reach a shared understanding of the patient's problem and a shared consideration of options for treatment. In the case of a magic performance, unspoken expectations are similar. In both, the performer is aware of the fragility of the relationship (Lamont and Wiseman, 1999).

Finally, though all stages of a consultation are important, the closing moments have a critical impact on a patient's retrospective perception. Shaping an audience's final impression is a key skill, but the significance of this phase is often overlooked. Clinicians are often preoccupied by the demands of the system in which they work, such as seeing a requisite number of patients, entering data onto the computer system and dealing with unexpected contingencies. Many consultations come to an abrupt halt without attending to this final impression. Magicians, on the other hand, are expert at constructing a shaped and constructed perception in their audience. This recollection may not correspond with what was actually said or done by the magician, since recollections are notoriously plastic and subject to subsequent refashioning. Comparing clinicians' and magicians' techniques for ending a performance offer a rich area of performance science enquiry.

In close-up magic and the clinical consultation, then, an apparently effortless social interaction conceals a complex “inner performance” where cognitive and fine motor skills are interwoven. This is an effortful process underpinned by years of preparation, prolonged practice and continual recalibration. As with other performances, each consultation requires the clinician to be functioning at multiple levels simultaneously, integrating the social practices of performance with the ability to draw upon canonical knowledge and “medical ways of thinking” (Prentice, 2013). This requires many functions to become automatized so that they can be subsumed into the wider performance without conscious effort or even awareness. Although the extensive literature on reflective practice (within and beyond medicine) addresses some of these issues, their implications have not been fully explored (Schon, 1987; de Cossart and Fish, 2005)

Although many consultations take place between one clinician and one patient, additional social, performative, and judgment skills are required when dealing with family or social groups. In some areas, such as pediatric surgery, this can be especially challenging. A clinician has to make an immediate assessment of complex family dynamics, connecting with adults and children at the same time and gathering information about relationships between them. This assessment requires continual review as the consultation evolves, and clumsiness can disrupt delicate dynamic structures. Magic performance requires similar skills.

This aspect of performance requires an acute sensitivity to social interactions, allowing a performer to “read” a situation within a few seconds. Close-up magicians recognize this and develop the ability to make and act on rapid social judgments. Performing successfully under such conditions involves recognizing when to step back as well as when to take center stage. Judgment is key, and such judgment only comes through long experience. (Magician 2's) adage that “A really good magician knows when not to perform” resonates with a dictum dinned into the author during his surgical training (and resonating with the maxim cited by Spary at the opening of this paper): “A surgeon knows how to operate. A good surgeon knows when to operate. And a really good surgeon knows when not to operate.” Clinicians have much to learn from magicians' specific techniques.

### Surgery as “reading bodies within a dextrous team”

The discussion so far has focused on interaction between clinician and patient. The focus during operative surgery is rather different, requiring a balance of individual and group skills within an ensemble. By framing surgery as a performance which requires “reading bodies within a dextrous team,” clear parallels with puppetry emerge. Operative surgery is a shared performance par excellence. It requires effective and often unspoken communication between its members. Ensuring that “transient teams” function effectively requires particular effort and preparation (Weldon et al., 2013, 2015a)

Members of a surgical team stand in extremely close proximity, their upper bodies often in contact while performing. At the same time, normal “social” modes of communication are suspended or distorted. Often there is little eye contact, as participants' attention is fixed upon a directly viewed organ or a screen. Faces may be concealed by masks and unnecessary speech is curtailed. Specialized abbreviated terminology (such as the names of instruments or anatomical structures) is widely used and the language of the operating theater is adapted for its purpose (Kneebone, 2014).

During an operation, members' ability to “read” one another's bodies is crucial. This is a subtle process, of which many participants are unaware. Much is conveyed by barely perceptible movements, tiny gestures take on a heightened significance, and sequences of movements are closely coordinated. For example, as a surgeon is tying a knot in a length of suture, other team members collectively but unconsciously anticipate the next move. The scrub nurse passes scissors to an assistant who reaches out to cut the excess thread, then hands the scissors back while the surgeon continues tying the next suture before the cycle repeats (Bezemer et al., 2011a,b, 2013, 2014; Cope et al., 2015).

Puppetry constitutes a performance domain where objects hold particular significance and offers illuminating parallels (Wilson and Milne, 2004; Francis, 2012). Puppeteers have an intense relationship with material objects, which they manipulate with a dexterity and precision which echoes the movements of the surgeon. Unlike the solo performance of a close-up magician, puppeteers are part of a theatrical ensemble which shares many of the constraints of the operating theater. Puppeteers perform in close physical proximity, especially when collaboratively working a single puppet. As with surgical teams, normal “social” modes of communication are suspended or distorted. Puppeteers cannot talk to one another during performance, and their collective focus is upon their puppets, not upon one another or the audience. Puppeteers deliberately foster a sensitivity to the physical signals they receive from one another while performing.

As described above, Bunraku puppetry requires coordinated teamwork similar to an operating team, where the roles of surgeon, first assistant and scrub nurse are distinct, complementary and interdependent and every member is indispensable. In surgery, the collective focus of the team is on the patient, much as the puppeteers' focus is the puppet. Although the operation is led by a principal surgeon (the “head” in Bunraku terms), at times the leadership role may switch within the team.

In surgery and in puppetry, fine motor skills (acquired individually but applied collectively) are displayed in a choreographed performance which is jointly constituted. The diversity of puppet performance offers parallels with open, laparoscopic and other types of surgery. Marionettes require puppeteers to handle long threads without getting them tangled up—a process well known to vascular surgeons when performing anastomosis (joining together) of small arteries and veins. Rod puppetry, like keyhole (laparoscopic) surgery, uses long rigid instruments to manipulate structures at a distance and requires similar levels of spatial awareness and precise control. For puppeteers, an intense focus on dexterity centers on the intersection between hands, instruments and materials and the ability to respond to subtle physical signals.

Surgery requires intense and prolonged practice over many years, leading eventually to unconscious mastery. Yet though dexterity and precision lie at the heart of operating, the aims of surgery (removing this tumor, repairing that injury, replacing this joint) overshadow the means by which this is achieved (the complex coordination of hands and fingers between members of an expert team). For many surgeons, their hands are a means of manipulating the instruments which effect the procedure rather than a focus of attention in their own right.

### Surgery as social performance

Though gaining such mastery is a satisfying experience in its own right, there are dangers than an unduly technicist focus will overshadow the social skills of live performance. This resonates with anxieties in the surgical world, where students and trainees may over-focus on technical challenges and lose sight of the wider clinical context.

In surgery, physical dexterity takes place within a team setting where surgeon, assistant(s) and scrub nurse work together as coordinated unit. In the past, stable teams of surgeons, nurses and others worked together for years or decades, developing shared vocabularies of embodied practice. The cultural landscape of contemporary surgery within the UK National Health Service is very different. Current practice often requires a surgical team to come together and immediately perform, even if its members have not met before. Although developments such as the World Health Organization checklist stipulate that team members be introduced to one another and review each forthcoming operation within a standard format, the focus is on the patient and relatively little attention is paid to collective preparation for the physicality of surgery (Lingard et al., 2005, 2008, 2011; Koschmann et al., 2007; Gawande, 2010; Koschman et al., 2011).

Puppetry has much to offer here. Like surgical teams, puppeteers often expected to form newly constituted groups composed of unfamiliar colleagues. Physical preparation plays an important role. Warming-up routines include muscle exercise, breathing and transfer exercises, where imaginary objects are “passed” between members of the team. Such preparatory exercises are seen as especially important in a performance genre where (as in surgery) fine motor control is of primary importance and where performers are expected to maintain unnatural postures for extended periods. This provides an interesting distinction from other types of performance.

In addition, the motor skills of puppetry place particular demands upon hands and fingers. Puppeteers have developed specific routines and approaches for developing their hand and finger skills (Video 3). The puppeteers in this study were astonished to discover that surgical teams do not warm up their hands in a similar way before surgery (despite the need to operate uninterruptedly for long periods) or pay attention to posture and the reduction of strain.

A further striking feature is the presence or absence of feedback and critique (within and outside the team) in routine performance. In surgery there is seldom a formalized framework for providing such feedback. In many teams, critique only takes place when something has gone wrong. Puppeteers, by contrast, expect and rely upon critique to improve and refine their work, perceiving it as entirely normal. This feedback seems especially relevant when the patient is present as audience during an operation. As outlined above, traditional framings of the operating theater—as a site where the “patient as person” vanishes for the duration of the procedure—no longer hold. The need to attend to the perceptions of the “patient as audience” can impose significant additional cognitive load on the surgical team, especially when technical complications arise. There is much to be learned about the “patient as audience” during procedures performed under local anesthetic.

## Discussion of methodology

This paper presents a comparative inter-practitioner study which goes beyond “traditional” observational approaches where a single domain of expert practice is the focus of a detached researcher's attention and where the researcher makes no claim to expertise in that domain. Here the boundaries between “research as observer” and “researcher as practitioner” become smudged. Indeed, the expectation in these case studies was that all participants would gain insight, new knowledge and “reciprocal illumination” which would enrich their practice (Kneebone, 2015). The paper does not attempt to generalize but recognizes the unique nature of the sample it presents. The experts it describes are all exceptional, both as performers in their individual fields and in having an interest in moving beyond their own frames to engage with the worlds of surgery and one another.

Thematic analysis was selected as a methodological approach in view of the exploratory nature of the enquiry. Strengths of this approach include its flexibility and usefulness in sense-making within a large data corpus, especially one that has emerged in a complex research environment (rather than being assembled to address a specific question or questions). This speaks to the definition of performance science set out in the paper's introduction, with its emphasis on a “multidisciplinary study which draws together methodologies across numerous scientific disciplines.” Challenges include the difficulties of dealing with a diverse and extensive data set, and the need to work within the constraints of access to busy professional people (surgeons and others) for whom such research is not a core activity.

The nature of the expert practices in this study presents additional challenges to the researcher. To some extent the hiddenness of surgery is addressed through simulation, inviting outsiders to participate in practices which are normally inaccessible. The study's use of simulation as a means of providing a shared experience of embodied practice within a closed clinical world proved of particular value with puppeteers. Yet however realistic such simulation may be, it remains a simulation and cannot capture the lived experience of actual surgery on real patients. There is much to be explored in characterizing the strengths and limitations of simulation as tool for cross-disciplinary research, though such exploration lies outside the scope of this paper. The secrecy of magic poses different challenges, since there is much about magicians' practice which they will not disclose to outsiders. Such differences must be taken into account when attempting to make comparisons.

## Conclusions

This paper started by locating surgical performance within an historical arc from the early eighteenth century (and earlier) to the present, suggesting that surgery be positioned alongside other instances of performance. Comparison with types of activity that are clearly identified as performance shows parallels and similarities that strengthen this claim.

The paper addresses the research questions as follows:

1. To what extent and in what ways can surgical practice (both consultation and operation) be considered as performance?

The paper concludes that surgery has a claim to be considered within the canon of performance science. The case studies support the proposition that surgery exhibits similarities with other domains of expert performance. Though no single parallel is exact or sufficient, selected examples use analogy to highlight the performative nature of surgery. By separating constituent elements of surgical practice (in this case the consultation and the operation), the study identifies cognate practices outside surgery for comparison. At the same time as identifying similarities, however, the study also highlights differences. These include the stakes for performers and audience, the different types of dexterity requirements and the characteristic composition of surgical teams.

2. How does comparison with two domains domains of non-surgical performance (close-up magic and puppetry) illuminate understanding of surgical practice as performance?

The two domains of non-surgical practice identify aspects of performance that focus attention on aspects of surgical practice that lie concealed when the spotlight is on diagnosis and treatment of disease. This comparison highlights close-up performance and dextrous teamwork as exploratory lenses for examining consultation and operative aspects of clinical practice.

3. In what ways might including surgery within the canon of performance studies enrich the field of performance science?

For performance science, surgery offers unexplored examples of comparative performance and potentially rich areas of research. Although this is a promising field, such studies are at an early stage and the impact of such study remains to be seen.

For experts in domains outside medicine (such as the magicians and puppeteers in this study), surgery provides an external frame that brings hidden aspects of their own practice into view, enabling them to refine their own practice through comparison with a cognate domain. Viewing their own practice with the “eyes of newness” invites them to foreground and articulate what may have become implicit and therefore inaccessible to themselves and others.

For clinicians, such study identifies aspects of their practice that can be addressed and improved, learning from the practices of others outside medicine. Engaging with the literature and collective experience of performance science provides access to perspectives that transcend the specifics of a given domain. For example, clinicians can learn much about becoming expert in social interaction through specific techniques for engaging audiences (in this case patients) in performance and making them feel involved and valued. These techniques can be (and are) taught and learned within magic, but are not taught and learned within surgery.

These case studies identify provisional categories of performance within surgery. Further work is needed to test, refine, and challenge such categorical relationships and to identify further instances which may confirm or disconfirm this framing. The paper provides preliminary evidence that surgery may usefully be considered as performance and that systematic exploration from a performance science perspective offers benefits to researchers, performers and clinicians.

## Author contributions

RK has conceived and written this paper.

### Conflict of interest statement

The author declares that the research was conducted in the absence of any commercial or financial relationships that could be construed as a potential conflict of interest.

## Footnotes

^1^http://journal.frontiersin.org/journal/psychology/section/performance-science/ (Accessed on August 23, 2016)

## Supplementary material

Video 1 **Surgeon controlling bleeding in simulated surgery**.

Video 2 **Public engagement simulation showing treatment of a stabbed patient**.

Video 3 **Puppetry finger exercises**.

Video 4 **Bunraku puppet slipping on ice**.
