# Supplementary material for: Performing Surgery: Commonalities with Performers Outside Medicine
Source: Front Psychol. 2016 Aug 31;7:1233. doi: 10.3389/fpsyg.2016.01233 (PMC5006638; doi:10.3389/fpsyg.2016.01233)
Supplement: Supplementary file 5 [file DataSheet1.DOCX]

**Appendix**

In the interests of brevity, the data presented in the Results section (above) has been selected from a wider set of thematically-grouped verbatim quotations which form part of the overall data set. The following quotations amplify or supplement that selection.

The following are grouped within the thematic structure used in the main body of the paper.

**Magic**

**1 Magic as social performance**

*Magician 4*: ‘We’re doing something secretly and presenting something overtly to the audience and trying to bridge that gap with an engaging presentation. […] If you sit in your room and practise a coin trick in front of someone over there who doesn’t exist, there’s no actual magic. There needs to be someone who misinterprets what they’ve seen you do before there is a trick.’

a) Gaining attention

*Magician 1:* ‘You need to enter a room with people in a way where you’re not threatening, but you can establish your presence there in a way that they feel comfortable with you.’

b) Maintaining attention

*Magician 2:* ‘In magic the face and the eyes are the most important. As a magician I need to know what’s happening with the eyes. I have to know what you’re thinking and to know that you know what I’m thinking at any moment. Because you get the effect of the magic from my attitude towards it - and the way you get my attitude is to be reading what I feel about it […] If I just look down at my paper and talk, I’ve got no idea where your attention is. But if I then look up at you - I can tell at once that I’ve got your attention. Can read your reaction and get instant feedback. By understanding where people’s attention is - and helping them understand, by guiding them - you can use the skills that we [meaning ‘we magicians’] do naturally, because we’ve studied them and understand why it works.’

c) Closing the performance

*Magician 1*: ‘Magicians use the word ‘misdirection’. But actually it is really directing. Beginning magicians think it’s about taking stuff away. But you can’t really hide stuff. What you can do is build things. Misdirection is an additive process. You add things to create an environment or a world within which magic can happen - as opposed to taking stuff away so you don’t see stuff. […] People think it’s about hiding things - it’s not, it’s about creating things.’

*Magician 2: ‘*I think what magicians are very good at doing is making the unimportant seem incredibly important and what’s absolutely critical very unimportant’.

*Magician 3:* ‘The magician is the honest liar. He tells you he’s going to do something that will fool you and then he does. If you [the magician] are just a smart aleck and you’re smarmy and showing off, that’s not very interesting magic. What you want to be doing is bringing people and say “I’m going to challenge your intellect, and I’m going to do something that’s going to defy any natural laws. But it’s going to be a conspiracy between you and me, kind of suspension of disbelief, and we’re both going to have to work on this to make it happen”. And if you blow it in some way - you lose trust, or they see what you’re doing - the bubble’s gone, it’s not going to work. It’s quite a delicate thing to have to do’.

**2 Magic as motor performance**

a) Gaining manipulative skills

*Magician 4:* ‘You have to take pleasure in the practice. A magician who specialises in sleight of hand will practise all the time’.

*Magician 4:* ‘This ability to apply fine motor skill within performance is something that only some magicians successfully master. They all start off with the manipulative skill - gained in isolation, through solitary practice (like medical students studying and memorising). But then they have to widen out and become performers. And this requires different kinds of skill’.

*Magician 2*: ‘Children now learn from Google. See someone from the elbows down. Learn incorrectly, from someone who has learned it third hand. [This] has created a generation of magicians with phenomenal technique, but the standard of magic has never been lower. No understanding of timing, performance skills, story telling. The story telling is the magic.’

**3 Magic as an internal process for the magician**

a) ‘The shift from you to them’

*Magician 5:* ‘Magic is about engaging with the magician, not the magic’.

*Magician 3:* ‘The reason why a lot of people don’t engage with magic is that they don’t engage with the magician, because the magician is putting this thing out in front of them, the effect, and thinking that’s what it’s about. It’s not about that’.

b) Awareness of and sensitivity to the audience

*Magician 3*: ‘You may have seen magicians coming round at parties, coming round entertaining groups of people having a drink or something like that. It would be completely crass in a normal situation to barge up to people and say ‘I want to show you this or that’, yet the magician seems to think he or she’s got a licence to do it. That’s a very delicate thing to work out. Maybe it’s the same sensitivity you need when dealing with patients.’

*Magician 2:* ‘As a performer, our radar in the first 10 seconds (as we walk out in front of the audience) is on maximum. Same as going to see the doctor’.

**Puppetry**

**1Puppetry as group performance**:

a) Establishing cohesion as a group

*Puppeteer 1:* And kind of a level of focus and tension, and how sort of - we have something in theatre called ‘circles of attention’, so that your focus might be this [gestures] sort of small circle around you but sometimes it might widen out. But you can control and jump between those according to … The puppet’s the main focus but I need to be aware that the audience at the back are laughing at something the puppet’s doing and I need to respond to that.

b) Group warm-up procedures

*Puppeteer 1*: They won’t not do it [warming up]. I mean, you can see the difference. You can feel the difference and you can see the difference. And the audience can see the difference. At the end of the day it’s about confidence as well.

*Puppeteer 1*: The first thing we do when we come together for the first time is very simply to say who we are and what our role is within the team.

*Puppeteer 5:*  ‘We’re making our hands move without thinking about it. Then ‘passing on’ a particular movement to the next person in line, who has to copy it. We’re letting it grow without thinking about it too much.’

*Puppeteer 1*: ‘They’re [puppeteers] very close together. In each other’s way, most of the time. They’re all looking at the puppet (as opposed to a patient). Their communication between them is not using eye contact. In our case not verbal. If the puppet is speaking, they’re not speaking to one another. If the puppet’s not speaking, there’re generally no words to express what needs to happen next. There’s an element of feeding off each other to work as a team, and sometimes having to improvise what to do or what needs to happen next.’

c) Expectations and role of critique and feedback.

*Puppeteer 3:* ‘There’s always feedback and critique - from the director especially, but also from colleagues, audience and critics.’

**2 Puppetry as motor performance:**

a) Manipulation of strings and rods

*Puppeteer 2*: ‘Puppetry is about “teaching your fingers to hear” - becoming sensitive to what is happening with the puppet’.

b) Finger and hand warm-up procedures

*Puppeteer 1:* ‘Most actors and dancers don’t have much focus on hands at all. They’ll focus on back or legs or whatever. But for puppeteers very particularly it’s hands […] Same way as a sportsperson will warm up for a race, so they don’t pull a muscle and they’re ready to perform. And in any kind of dance or gymnastics, you are stretching the range of ability that those muscles can do. So doing those muscles in terms of the individual fingers means that we’ve got more dexterity.
